# Supplementary material for: COVID-19 Adenoviral Vector Vaccination Elicits a Robust Memory B Cell Response with the Capacity to Recognize Omicron BA.2 and BA.5 Variants
Source: J Clin Immunol. 2023 Jun 16;43(7):1506–18. doi: 10.1007/s10875-023-01527-2 (PMC10499924; doi:10.1007/s10875-023-01527-2)
Supplement: Supplementary file 1 — Supplementary file1 (PDF 1036 KB) [file 10875_2023_1527_MOESM1_ESM.pdf]

Supplementary Tables (n=3) and Figures (n=6)

Supplementary Table 1: Participant characteristics

**Participant number**	**Age at inclusion (year)**	**Sex (M/F)**	**Time between doses of ChAdOx1 (days)**
1	26	M	84
2	27	F	84
3	29	F	84
4	32	F	78
5	33	F	83
6	33	F	84
7	33	M	84
8	34	F	82
9	35	M	84
10	36	F	84
11	37	F	83
12	37	F	84
13	38	F	84
14	42	M	84
15	43	F	84
16	45	F	84
17	45	F	84
18	47	F	76
19	48	F	88
20	48	F	83
21	48	F	85
22	50	F	79
23	54	F	84
24	56	F	91
25	57	M	84
26	58	F	84
27	58	F	95
28	59	F	70
29	61	M	84
30	64	M	84
31	65	M	84
	Median: 45 (range: 26-65 years)	74% female	Median: 84 (range: 70-95 days)

Supplementary Table 2: Flow cytometry antibody panel composition

Tube	Fluorochrome															
	BUV395	BUV496	BUV737	BV421	BV480	BV650	BV711	BV786	FITC	BB700/PerCP Cy-5.5	PE	PE-Vio615	PC7/ PE-Cy7	APC	AF700	APC-H7/ APC-Cy7
**1. Trucount**	—	—	—	—	—	—	—	—	CD3	CD45	CD16+ CD56	—	CD4	CD19	—	CD8a
**2. SARS-CoV-2-specific Bmem**	WH1 RBD	CD3	WH1 RBD	CD27	BA.2 RBD	BA.5 RBD	CD21	CD71	IgG2+ IgG3	IgD	IgG1+ IgG2	IgA	CD19	IgG4	Fixable viability	CD38
**3. Streptavidin control**	Streptavidin	—	Streptavidin	CD27	Streptavidin	Streptavidin	—	—	CD3	IgD	—	—	CD19	—	Fixable viability	—

Supplementary Table 3: Details of antibodies used in flow cytometry panels

**Marker**	**Fluorochrome**	**Clone**	**Vendor**	**Cat. number**	**Volume /test (µL)**	**Tube**
CD3	BUV496	UCHT1	BD Biosciences	612940	1	2
CD3	FITC	UCHT1	BD Biosciences	555332	1	3
CD3	FITC	SK7	BD Biosciences	662995*	46ng	1
CD4	PE-Cy7	SK3	BD Biosciences	662995*	30ng	1
CD8a	APC-Cy7	SK1	BD Biosciences	662995*	126ng	1
CD16	PE	B73.1	BD Biosciences	662995*	33ng	1
CD19	APC	SJ25C1	BD Biosciences	662995*	46ng	1
CD19	PE-Cy7	SJ25C1	BD Biosciences	557835	5	2, 3
CD21	BV711	B-ly4	BD Biosciences	563163	5	2
CD27	BV421	M-T271	BD Biosciences	562513	1	2, 3
CD38	APC-H7	HB7	BD Biosciences	656646	1	2
CD45	PerCP Cy-5.5	2D1	BD Biosciences	662995*	120ng	1
CD56	PE	NCAM16.2	BD Biosciences	662995*	22ng	1
CD71	BV786	M-A712	BD Biosciences	563768	1	2
Fixable viability	AF700	N/A	BD Biosciences	564997	0.1	2, 3
IgA	PE-Vio615	REA1014	Miltenyi Biotec	130-116-882	1.5	2
IgD	BB700	IA6-2	BD Biosciences	566538	1	2, 3
IgG1	PE	G17-1	BD Biosciences	624049	0.1	2
IgG2	FITC	HP6002	BD Biosciences	624045	0.5	2
IgG2	PE	HP6002	BD Biosciences	624049	1	2
IgG3	FITC	HP6047	BD Biosciences	624045	0.5	2
IgG4	APC	SAG4	Cytognos	CYT-IGG4AP	2	2
Streptavidin	BUV395	-	BD Biosciences	564176	0.67	2, 3
Streptavidin	BUV737	-	BD Biosciences	612775	0.67	2, 3
Streptavidin	BV480	-	BD Biosciences	564876	0.67	2, 3
Streptavidin	BV650	-	BioLegend	563855	0.13	2, 3
*Antibodies part of the Multitest™ 6-color TBNK kit (BD Biosciences)						

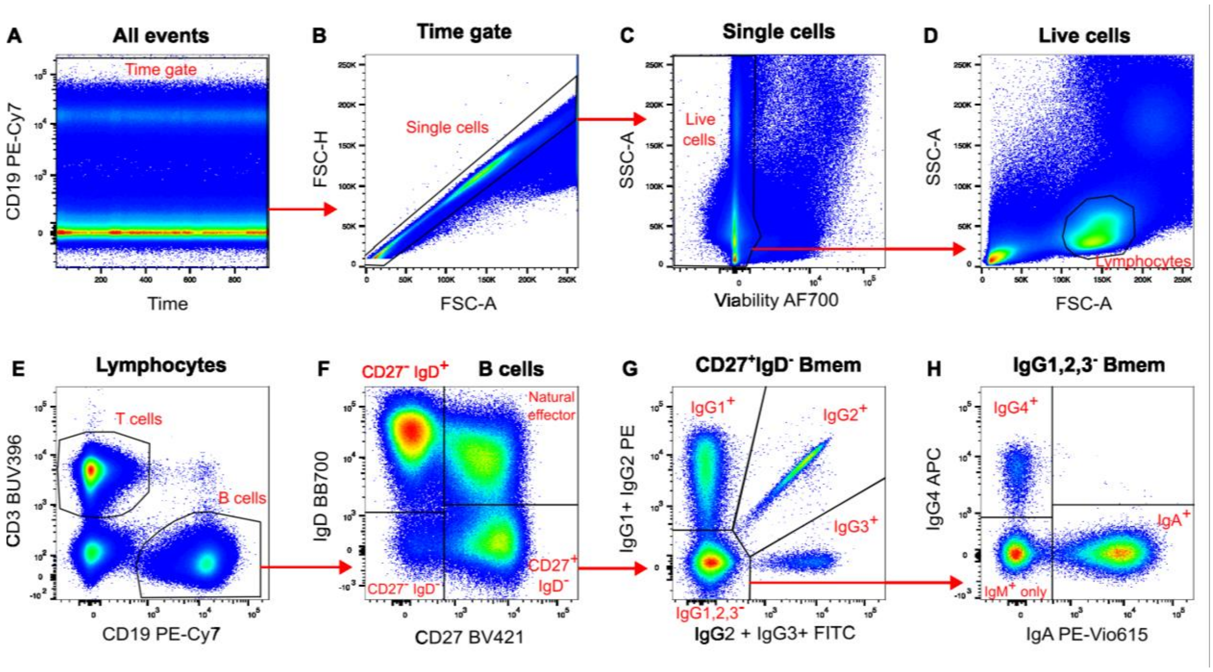

Supplementary Fig 1: B-cell gating strategy. (**A**) CD19 vs Time was used to ensure a steady signal of CD19 PE-Cy7. (**B**) Doublets were excluded by gating on FSC-A vs FSC-H. (**C**) Dead cells were excluded by gating on SSC-A vs Viability. (**D**) Live cells were gated for SSC^{lo} lymphocytes. (**E**) Lymphocytes were gated for $CD3^{+}CD19^{-}$ T cells and $CD3^{-}CD19^{+}$ B cells. (**F**) B cells were gated for $CD27^{-}IgD^{+}$ naive B cells and $CD27^{+}IgD^{+}$, $CD27^{+}IgD^{-}$, and $CD27^{-}IgD^{-}$ Bmem subsets. (**G**) Each Bmem subset was gated for $IgG1^{+}$, $IgG2^{+}$, and $IgG3^{+}$ populations, then (**H**) the triple negative population was further divided into $IgG4^{+}$, IgA^{+}, and IgM^{+} only cells. Representative plots from donor sample taken post-dose two of ChAdOx1.

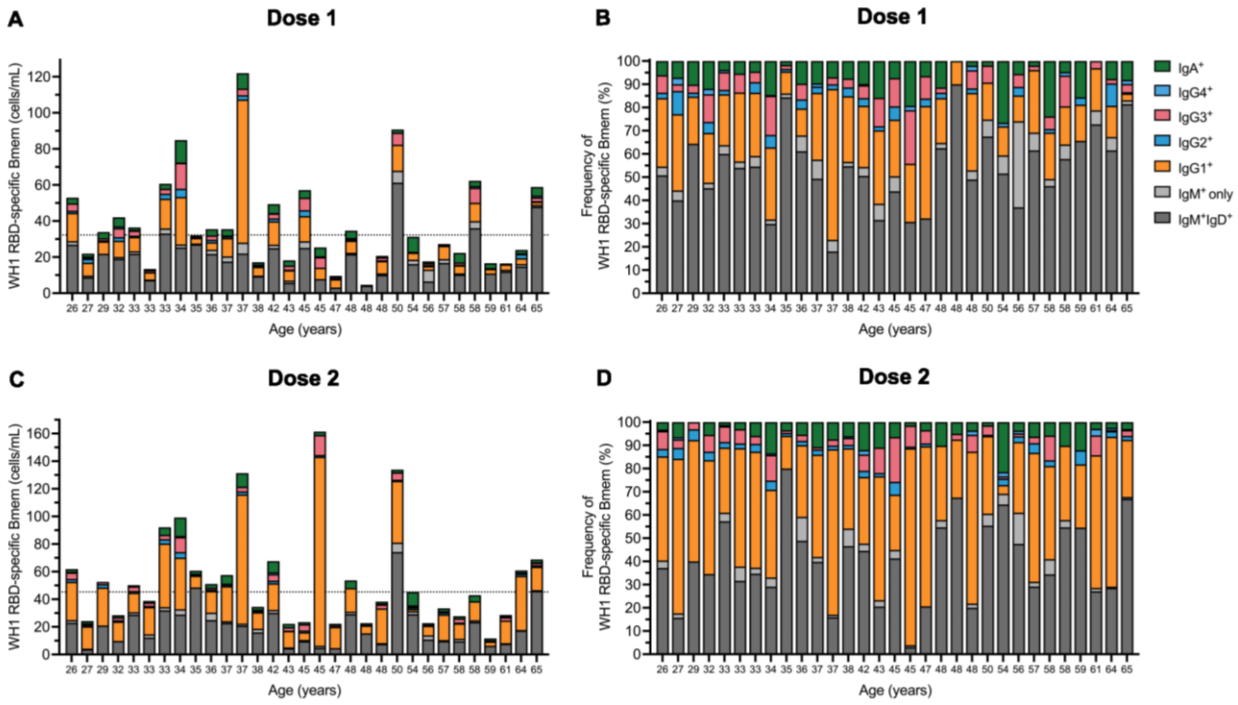

Supplementary Fig 2: Ig isotype and IgG subclass distribution in individual donors. (**A**) Absolute numbers and (**B**) relative distribution of WH1 RBD-specific Bmem expressing IgG1, IgG2, IgG3, IgG4, IgA, IgM only, or IgM and IgD post-dose one of ChAdOx1. (**C**) Absolute numbers and (**D**) relative distribution of each Ig isotype and IgG subclass post-dose two of ChAdOx1. Dotted lines represent median WH1 RBD-specific Bmem numbers.

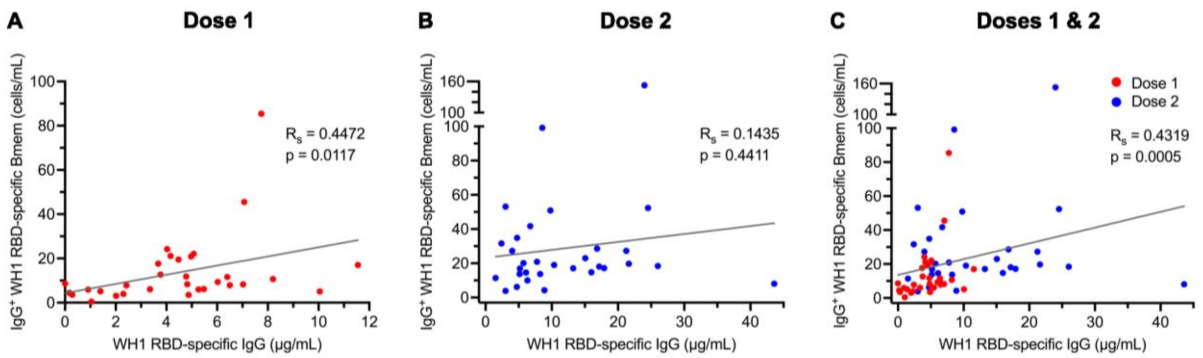

Supplementary Fig 3: Correlation between IgG+ WH1 RBD-specific Bmem and plasma IgG. Correlation between absolute numbers of IgG+ WH1 RBD-specific Bmem and WH1 RBD-specific plasma IgG (A) post-dose one, (B) post-dose two, and (C) combined post-dose one and two of ChAdOx1. Non-parametric Spearman's rank correlation (R_S), solid line represents simple linear regression line.

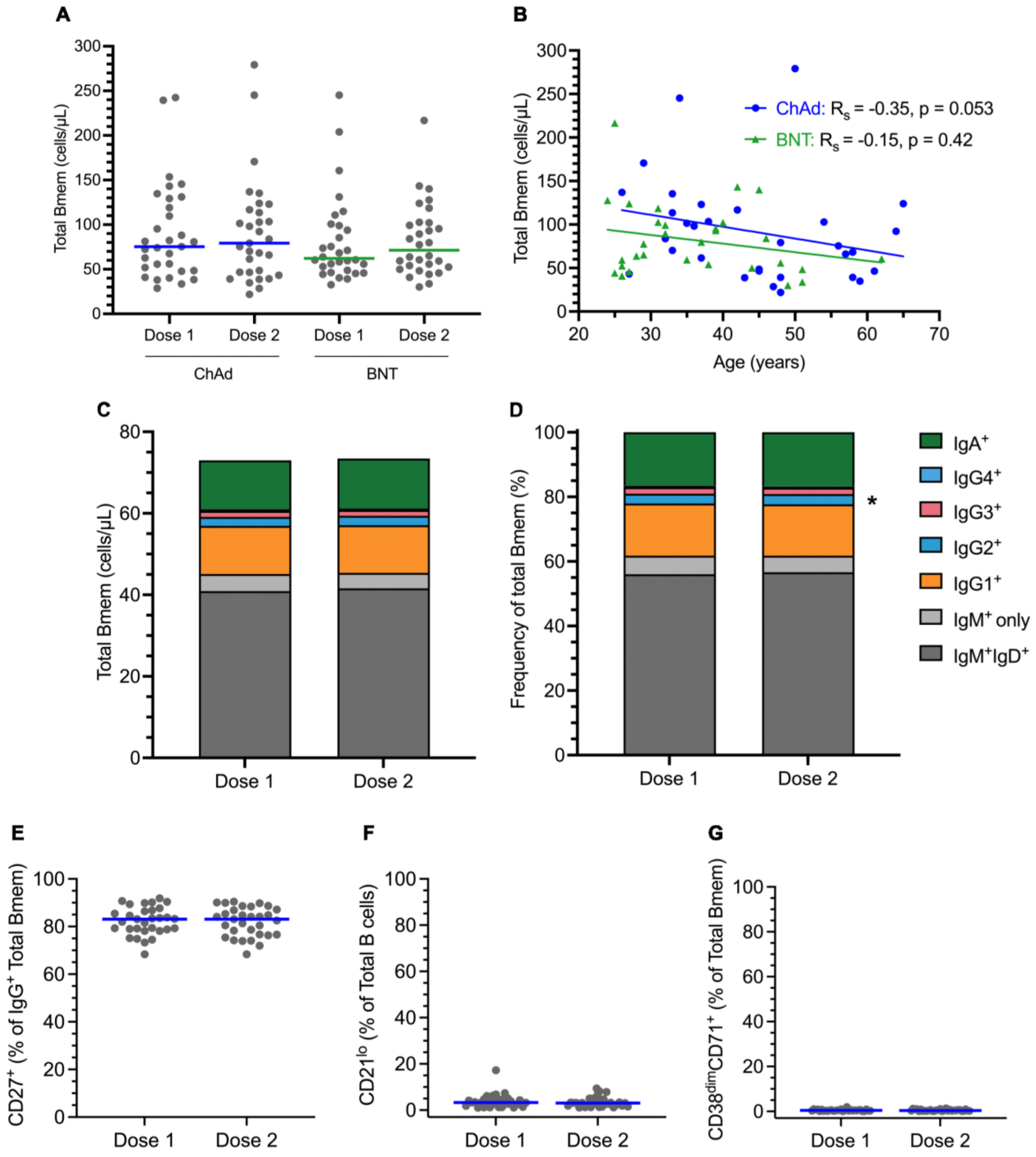

Supplementary Fig 4: Immunophenotype of total Bmem following ChAdOx1 vaccination. (**A**) Absolute numbers of total Bmem four weeks post-dose one (Dose 1) and four weeks post-dose two (Dose 2) of ChAdOx1 (ChAd) and BNT162b2 (BNT). (**B**) Correlation of total Bmem with age after either ChAdOx1 or BNT162b2 two-dose vaccination. Non-parametric Spearman's rank correlation (R_S), solid line represents simple linear regression line. (**C**) The percentage of total Bmem expressing IgM only, IgM and IgD, IgG1, IgG2, IgG3, IgG4, or IgA four weeks post-dose one (Dose 1) and four weeks post-dose two (Dose 2) of ChAdOx1. (**D**) The percentage of WH1 RBD-specific Bmem expressing each Ig isotype and IgG subclass postvaccination with ChAdOx1. (**E**) Frequencies of total IgG^+ Bmem expressing CD27 post-vaccination with ChAdOx1. (**F-G**) The frequencies of total (**F**) $CD21^{lo}$ and (**G**) $CD38^{dim}CD71^+$ Bmem post-vaccination with ChAdOx1. ChAdOx1 n=31, BNT162b2 n=30. Wilcoxon matched-pairs singed rank test. Only significant differences shown. *p<0.05, ****p<0.0001. BNT162b2 data previously published.[1]

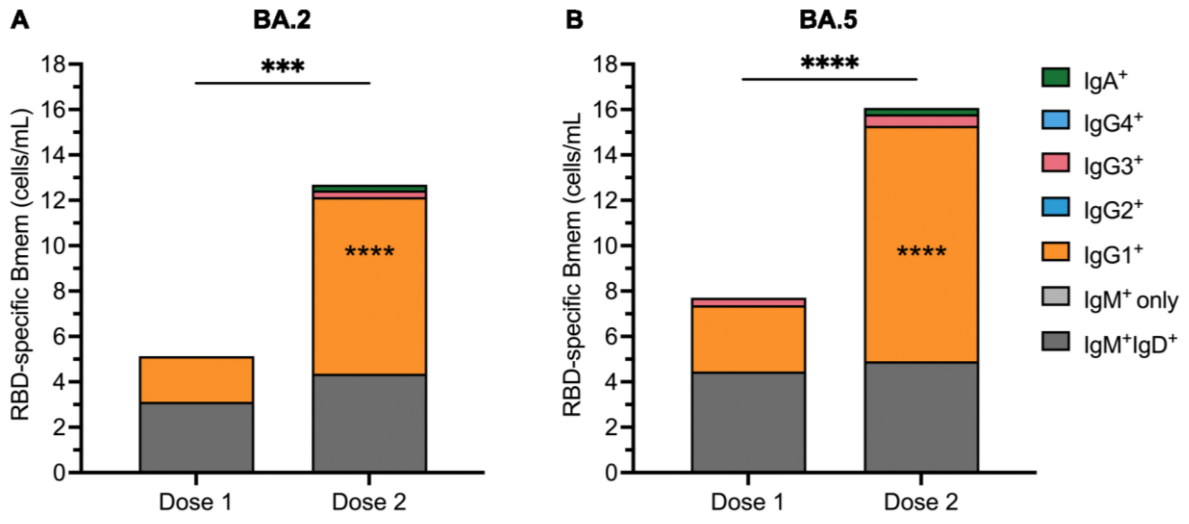

Supplementary Fig 5: Relative distribution of the Ig isotypes and IgG subclasses of BA.2 and BA.5 RBD-specific Bmem following ChAdOx1 vaccination. (**A**) The percentage of RBD-specific Bmem binding BA.2 expressing IgM only, IgM and IgD, IgG1, IgG2, IgG3, IgG4, or IgA four weeks post-dose one (Dose 1) and four weeks post-dose two (Dose 2) of ChAdOx1. (**B**) The percentage of RBD-specific Bmem binding BA.5 Bmem expressing each Ig isotype and IgG subclass postvaccination with ChAdOx1. n=31. Wilcoxon matched-pairs singed rank test. Only significant differences shown. ***p<0.001, ****p<0.0001.

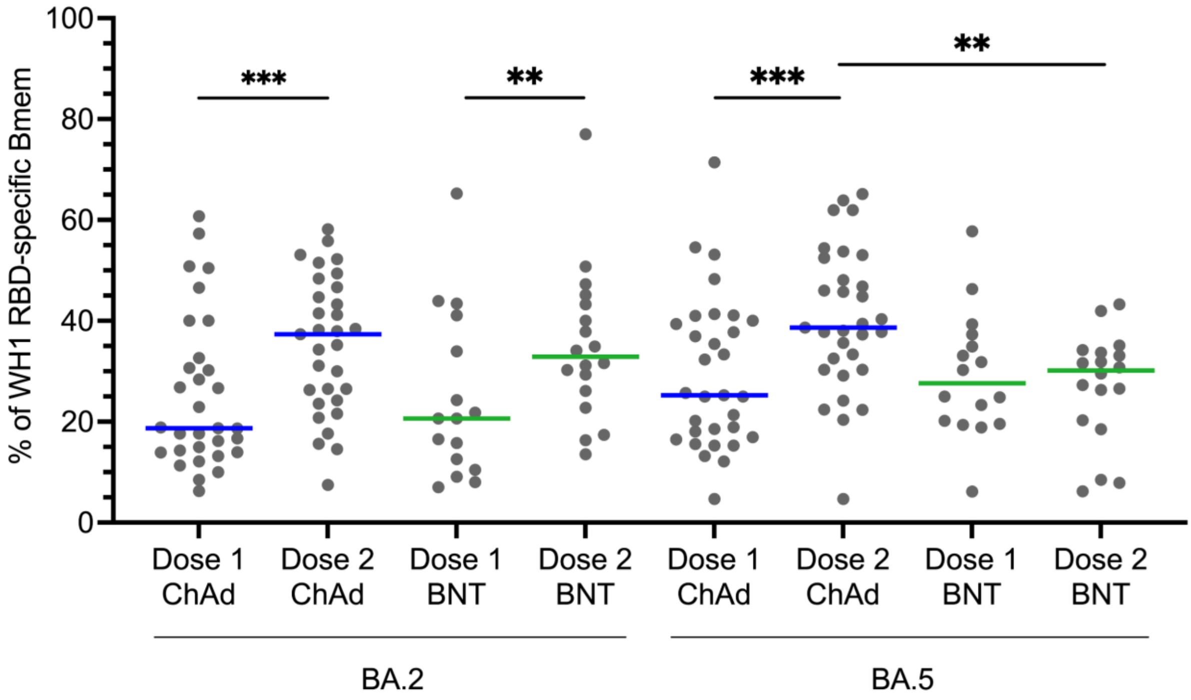

Supplementary Fig 6: Comparison of the capacity of WH1 RBD-specific Bmem to bind Omicron BA.2 and BA.5 after vaccination with ChAdOx1 or BNT162b2. Frequencies of WH1 RBD-specific Bmem that bound BA.2 and BA.5 RBD post-vaccination with ChAdOx1 (ChAd, n=31) or BNT162b2 (BNT, n=16 post-dose one and n=18 post-dose two). Solid lines indicate median values. Mann-Whitney test for unpaired data and Wilcoxon matched-pairs signed rank test for paired data. Only significant differences shown. **$p<0.01$, ***$p<0.001$. BNT162b2 data previously published [1].

Reference:

1. Hartley GE, Edwards ESJ, Varese N, Boo I, Aui PM, Bornheimer SJ, *et al.* The second COVID-19 mRNA vaccine dose enhances the capacity of Spike-specific memory B cells to bind Omicron BA.2. *Allergy* 2022. https://doi.org/https://doi.org/10.1111/all.15624
